# Synergistic Multi-Model Fusion for Efficient–Accurate Multi-Defect Detection in Power Lines

**DOI:** 10.3390/s26103185

**Published:** 2026-05-18

**Authors:** Linfeng Xi, Tao Shen, Guanglong Zhao, Nan Wang, Zhi Li

**Affiliations:** 1 School of Electrical Engineering, University of Jinan, Jinan 250022, China; 2 School of Control Science and Engineering, Shandong University, Jinan 250061, China

**Keywords:** UAV power line inspection, multi-target defect detection, synergistic multi-model fusion, C3_Mamba

## Abstract

In unmanned aerial vehicle (UAV)-based power line inspection, multi-scale defects and complex backgrounds challenge the balance between detection accuracy, speed, and model lightweighting, limiting automated grid inspection. This paper proposes a Multi-Scale Mamba Framework (MS-Mamba) for efficient and accurate defect perception. A drone inspection dataset containing 5137 images from 14 defect categories was constructed and divided into training and validation sets with an 8:2 split. To address the large scale variation among defects, the categories are decoupled into macroscopic, mesoscopic, and microscopic groups according to physical attributes and visual scales. As the core perception engine, a lightweight state-space mechanism is designed to balance accuracy and deployability. A spatial resolution-aware hierarchical reconstruction strategy and a dynamic feature selection mechanism are integrated to enhance feature extraction, reduce background redundancy, and improve small-target representation. Compared with the YOLOv5s baseline, MS-Mamba achieves an mAP@0.5 of 0.749, corresponding to a 15.6 percentage-point improvement, while reducing parameters by 0.13 M and computational cost by 1.7 GFLOPs. Ablation studies and visual analyses further confirm fewer missed and false detections in complex backgrounds. The developed end-to-end inspection system was validated through closed-loop engineering tests, demonstrating strong potential for industrial deployment.

## 1. Introduction

As the end power supply link connecting the power grid to millions of households, 10 kV distribution lines are a crucial cornerstone for ensuring residential power supply and the stable operation of the national economy [1]. Widely deployed across urban and rural areas, these lines are persistently exposed to harsh natural conditions (e.g., rainstorms, lightning) and vulnerable to external hazards like physical impacts and vegetation interference [2]. Any minor defects (e.g., bolt loosening, pin detachment) can trigger cascading faults such as line short-circuits and ground faults, causing large-scale regional power outages and even severe safety accidents, including fires and electric shocks [2,3]. Unmanned aerial vehicle (UAV) aerial photography has now replaced traditional manual inspection, greatly enhancing the efficiency and operational safety of distribution line inspections [4,5]. Additionally, the development of multisensor information fusion and robust state estimation has further promoted intelligent monitoring in power-related systems, improving the reliability, precision and robustness of system perception under uncertainty [6]. However, defect interpretation of inspection images still relies heavily on manual visual assessment, which is inefficient and labor-intensive. Detection results are also susceptible to subjective factors such as inspectors’ experience and fatigue, leading to high rates of missed and false detections [7,8]. Therefore, developing AI-based automated defect detection technologies to realize intelligent analysis and accurate identification of defects in UAV inspection images is key to advancing the intelligent and unmanned operation and maintenance of distribution lines, and an inevitable trend when aiming to achieve safe and efficient power system operation and maintenance [9,10].

**Figure 1:**
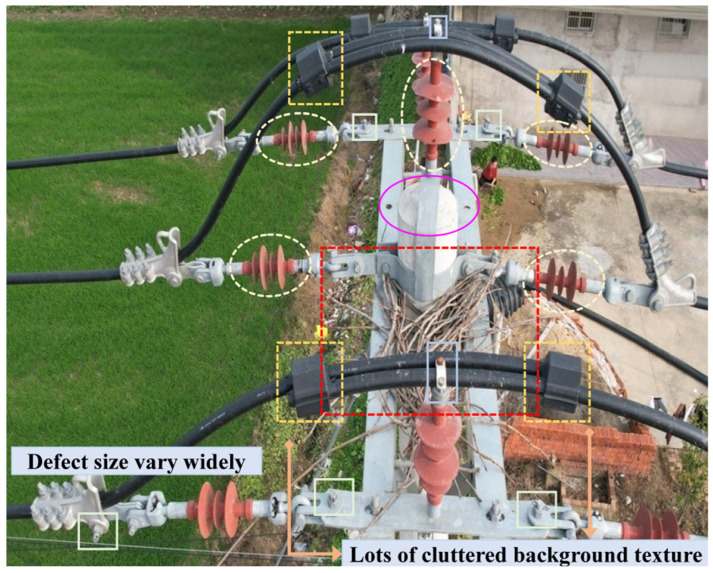
Representative defect locations and visual challenges in 10 kV power line defect detection, including scale variation and background interference.

Single-stage detection algorithms have become the mainstream solution for real-time power inspection due to their advantages of end-to-end inference and fast processing speed. For the detection of bird nests, Ma et al. proposed MID-Net, a multi-scale network [3]. For identifying bolt loosening, Lei et al. proposed a vision-based method that detects anti-loosening marking lines [11]. To address the challenge of detecting small-sized targets like bolts, Liu et al. proposed KCDet, a keypoint clustering detector [12]. However, in actual power line inspection scenarios, as shown in Figure 1, defect detection must handle multi-scale and multi-form targets. Figure 1 shows representative defect locations and highlights two key challenges: large scale variation and background interference. Defects range from macroscopic abnormalities, such as bird nests and foreign objects, to microscopic defects, such as missing pins, while dense fittings and complex backgrounds further complicate accurate recognition. Studies on visual recognition have also shown that fixed-scale feature representation or matching may suffer from scale inconsistency, especially when target proportions vary significantly across complex scenes [13]. Under a single model architecture, the feature learning of defects with different physical characteristics is prone to mutual interference, making it difficult to achieve the simultaneous detection of multiple target defects [14]. UAV-assisted power line inspection research has also shown that severe class imbalance, complex environmental interference, and collaborative recognition of multiple defect types remain major bottlenecks for automated defect detection [15]. Additionally, due to the perspective characteristics of high-altitude aerial photography, critical tiny defects such as missing bolts and pins occupy an extremely low pixel proportion in the images, with weak visual features that are easily overwhelmed by complex natural backgrounds. Moreover, inspection images are mostly in high-resolution formats, while the computational power of deployment platforms like drones and edge computing boxes is limited. The detection model must simultaneously meet the engineering requirements for both multi-class defect recognition accuracy and real-time processing [16].

For the detection of tiny defects in power inspection—characterized by low pixel ratios, faint features and easy occlusion by complex backgrounds—existing studies have conducted targeted explorations from the perspectives of cascaded architecture design, dedicated detector development and feature module optimization. Liu et al. [17] proposed a cascaded model based on improved YOLOv5, adopting a two-stage strategy of first detecting power line fittings and then performing refined recognition within fitting regions to detect tiny defects such as pin loss. Liang et al. [18] designed an FS-SSD detector integrating feature scaling and spatial context analysis, adapted to the detection of medium and small power targets in UAV images. Jiao et al. [19] constructed the MARF-CCN model by embedding hybrid attention and a cascaded classification network into Faster R-CNN, which improves the classification reliability of tiny bolt targets. Hu et al. [20] proposed DGW-YOLOv8, integrating deformable convolution and global attention into the backbone and combining the WIoU v3 loss to enhance the model’s training robustness on low-quality images. Chen et al. [21] proposed an insulator string defect detection method based on RGB color analysis and multi-scale feature compensation, which employs multi-level detection heads to compensate for missing high- and low-frequency information and improve fine-grained defect recognition under complex UAV inspection scenes. Although these methods significantly improve the recognition rate of single-type tiny defects, they mostly rely on single-model feature enhancement strategies and lack verification of simultaneous detection performance for multi-scale and multi-target defects. In addition, some methods cause a sharp surge in computational complexity due to complex modules, while others require the manual setting of key parameters, which sacrifices detection real-time performance and impairs the engineering practicability of edge deployment.

For the synergistic detection of multi-scale and multi-target defects, researchers have innovated algorithms tailored to the detection requirements of multiple defect types in distribution lines. He et al. [22] proposed the MFI-YOLO algorithm, improving YOLOv8 to strengthen the feature-learning capabilities for various insulator faults. Yi et al. [23] constructed PSTL-Net, which integrates a self-texture learning module and a patch-aware spatial attention module to realize the simultaneous localization and recognition of multiple components and defects in transmission lines. Zhang et al. [24] designed an FPF-focused perception framework, reconstructing the visual perception process via cross-scale attention guidance and Gaussian-guided activation supervision to solve the problem of tiny defect signals being submerged by complex backgrounds. Jiao et al. [25] proposed the YOLO-DTAD model, which adaptively allocates network resources through a dynamic task alignment mechanism to alleviate multi-task learning conflicts and realize the synergistic detection of multiple power defect categories. However, most existing multi-target detection methods adopt a serial cascading mode and lack adaptive grouping strategies based on defect physical attributes. This not only leads to low inference efficiency but also insufficient synergistic optimization among defect detection tasks, making it hard to meet the real-time detection requirements of practical inspections.

Given the computational resource constraints of edge devices and the need to balance detection accuracy and real-time performance, research on lightweight detection models has steadily advanced. Researchers have reduced computational overhead while retaining detection performance via backbone lightweighting and feature module improvement. Liu and Jiang [26] proposed LA-YOLO, combining a fast C2f backbone, an adaptive FPN and a decoupled detection head to achieve the efficient detection of small-sized insulator faults. Wu et al. [27] designed an enhanced lightweight model based on YOLOv8n and proposed MBLEConv to strengthen global information modeling, adapted to defect detection in insulators and vibration dampers. Tan et al. [28] adopted MobileNetV3 as the backbone, integrated SimAM attention and designed a lightweight CSPPC module to optimize feature fusion, reducing the model’s computational load while maintaining detection accuracy. Beyond conventional CNN- and YOLO-based designs, visual state-space models have recently been explored for high-resolution remote sensing object detection. Xiao et al. [29] proposed OriMamba, which uses Mamba-based state-space modeling to capture long-range contextual dependencies and enhance multi-scale feature representation under complex backgrounds. Nevertheless, a single lightweight model struggles to balance the detection performance of all targets when facing distribution line defects with significant differences in physical characteristics and scales (e.g., macroscopic foreign objects and microscopic missing parts). In addition, existing lightweight models lack adaptive feature learning and task decoupling mechanisms, with all defects sharing the same feature extraction and decision-making process. This makes customized optimization for different defect characteristics impossible, failing to meet the requirements of multi-defect collaborative detection.

To address this, we propose a synergistic multi-model fusion framework that follows the principle of divide-and-conquer and collaborative optimization: defects are grouped based on their physical attributes, with dedicated lightweight sub-models designed for each group to decouple learning interference and enhance specialized detection performance. Meanwhile, a C3_Mamba backbone network with linear complexity is introduced to reduce the overhead of multi-model execution. The system is integrated into a software platform supporting one-click processing, automatic annotation, and report generation, forming a complete closed loop from algorithmic development to industrial application. The main contributions of this paper are as follows:

1. A synergistic multi-model fusion framework integrating physical attribute grouping and Mamba is proposed, which effectively resolves the accuracy–efficiency trade-off dilemma in multi-scale power defect recognition.
2. Accurate and robust identification of 14 categories of power defects ranging from macro- to micro-scales is realized, which significantly strengthens the ability to discriminate tiny targets against complex background interference.
3. A C3_Mamba visual backbone network with linear complexity was developed, incorporating a spatial resolution-aware hierarchical reconstruction strategy and a dynamic selection mechanism, and thereby providing a lightweight solution for the rapid processing of UAV inspection.
4. An end-to-end power inspection system from intelligent perception to decision output is constructed, which achieves automatic defect recognition, classification and report generation, and completes engineering verification and closed-loop validation of applications.

The remainder of this paper is structured as follows. Section 2 elaborates on the proposed Multi-Scale Mamba Framework (MS-Mamba) and the design of its core perception network. Section 3 introduces the experimental setup, dataset, and evaluation metrics. Section 4 presents the experimental results with an in-depth analysis and discussion. Finally, Section 5 concludes this paper.

## 2. Proposed Methodology

### 2.1. Overall Architecture

**Figure 2:**
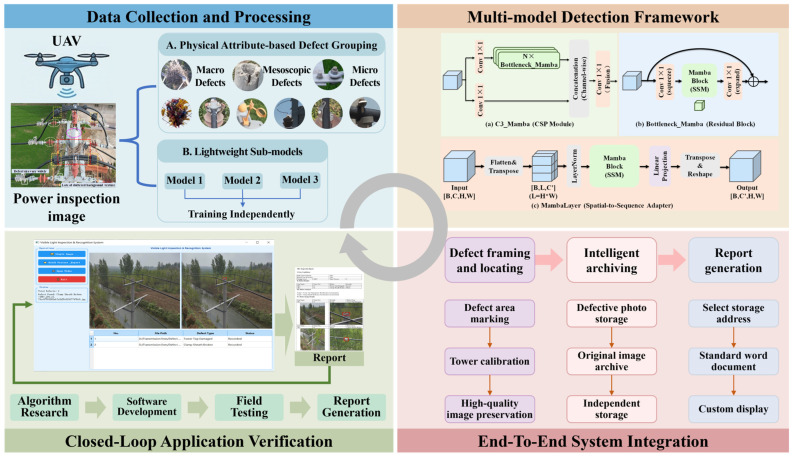
Overall architecture of the proposed synergistic multi-model defect detection system.

This paper proposes a multi-objective defect closed-loop detection system framework based on synergistic multi-model fusion. As shown in Figure 2, the overall workflow consists of four core stages.

First, in the data acquisition and processing stage, images captured by UAVs are classified using a physical attribute-based grouping strategy, which decouples diverse defects into three major categories: macro, meso, and micro. Three lightweight sub-models are trained separately for each category. Second, in the multi-model detection stage, the backbone network adopts the C3_Mamba module, integrating a spatial resolution-aware hierarchical reconstruction strategy and a dynamic selection mechanism to realize model lightweighting, precise feature extraction and accurate detection.

During inference, each UAV inspection image is sent to the three trained sub-models, which are responsible for macroscopic, mesoscopic, and microscopic defects, respectively. Each sub-model first removes low-confidence boxes and then applies NMS within its own detection results to reduce repeated predictions. After that, the valid results from the three sub-models are checked together. If any sub-model detects at least one valid defect, the image is regarded as defective, and the corresponding bounding boxes, defect classes, and confidence scores are output. If none of the three sub-models detects a valid defect, the image is regarded as defect-free. When different sub-models produce overlapping boxes in the same region, the box with the higher confidence score is kept, while boxes in different regions are retained as separate defect results.

Then, in the end-to-end system integration stage, an automated workflow covering defect localization, intelligent archiving and standardized report generation is constructed. Finally, in the application verification stage, the proposed algorithm is embedded into a self-developed inspection system to complete full-process verification from algorithm research, software development and real-scenario testing to report generation.

### 2.2. MS-Mamba Framework Architecture and Spatial Resolution Perception Mechanism

**Figure 3:**
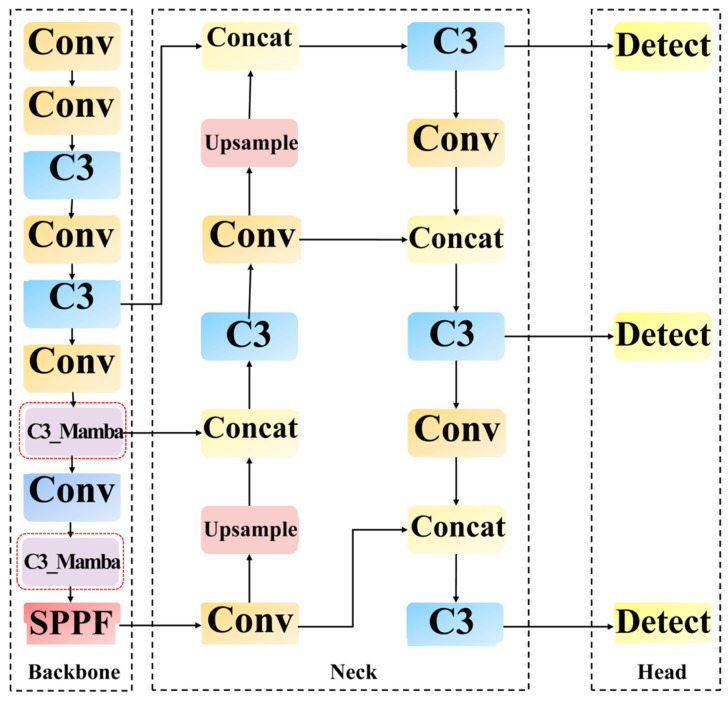
Overall network architecture of the MS-Mamba.

Owing to its highly modular architectural design and stable engineering implementation, YOLOv5s has emerged as the preferred baseline framework for numerous industrial vision tasks [30]. In light of this, this paper adopts YOLOv5s as the foundational architecture to construct the proposed MS-Mamba, tailored to power line inspection tasks. The overall network architecture is illustrated in Figure 3. This architecture comprises three primary components, the Backbone, the Neck, and the Head, which collaborate to accomplish feature extraction, fusion, and prediction. However, in UAV power line inspections, micro-defects such as missing split pins and loose bolts are highly susceptible to being obscured by cluttered field backgrounds. Constrained by their local receptive fields, traditional Convolutional Neural Networks (CNN) struggle to effectively model the global positional dependencies between these micro-defects and macroscopic power components, thereby failing to achieve precise identification.

To overcome this challenge, this paper improves the backbone network by proposing a spatial resolution-aware hierarchical reconstruction strategy. Specifically, this strategy dynamically allocates tasks based on the resolution of the feature maps. In the shallow layers of the network, pure convolutional modules are fully retained, leveraging their low computational overhead to efficiently extract local textures and edge contours from high-resolution images. Conversely, in the deep stages where semantic information is highly abstract—as indicated by the red dashed boxes in Figure 3—the original C3 structures are replaced by the proposed C3_Mamba modules to achieve long-range dependency modeling of global contextual information. The core rationale behind this hierarchical perception design is twofold: introducing the State Space Model (SSM) into the low-resolution deep feature maps accurately establishes the global connections between micro-defects and the macroscopic background, thereby enabling the detection of minute targets amidst cluttered backgrounds; simultaneously, refraining from applying complex global operators on shallow, high-resolution feature maps circumvents the consequent surge in computational cost. Ultimately, the conventional convolutions in the shallow layers and the C3_Mamba modules in the deep layers constitute a highly efficient and complementary feature extraction system, achieving an optimal balance between detection accuracy and computational load while ensuring real-time inference speeds on edge devices.

### 2.3. C3_Mamba Module

**Figure 4:**
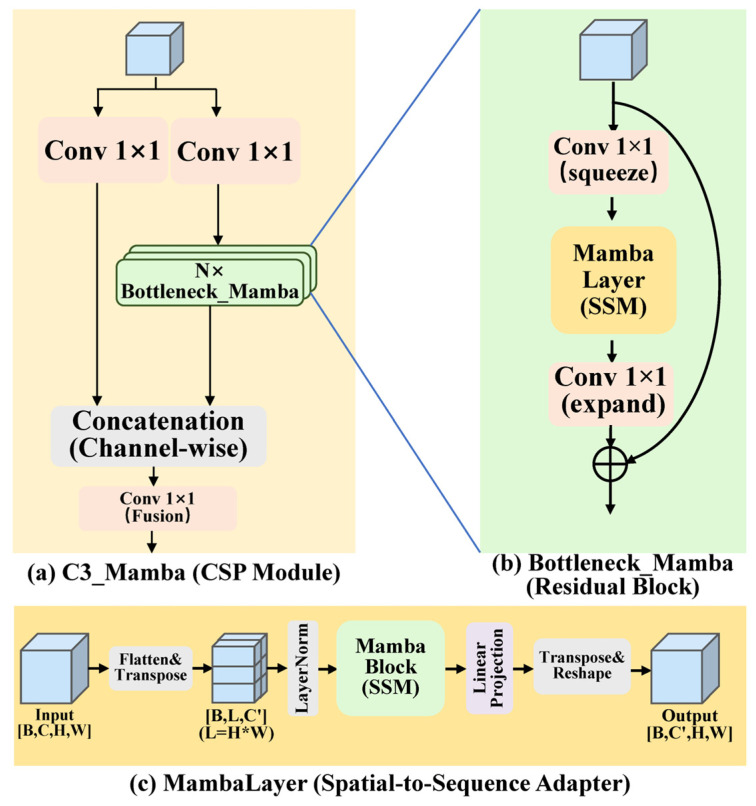
C3_Mamba module and its component architecture.

The complete outer-to-inner architecture of the progressively nested C3_Mamba module is illustrated in Figure 4. As illustrated in Figure 4a, C3_Mamba improves upon the dual-branch structure of Cross Stage Partial Network (CSPNet), where the main branch consists of multiple Bottleneck_Mamba components. As shown in Figure 4b, each Bottleneck_Mamba adopts a “compression–global perception–expansion” residual bottleneck architecture with an embedded MambaLayer. Within the MambaLayer (Figure 4c), features are transformed into a one-dimensional sequence to enable global information interaction, overcoming the local receptive field limitations of traditional 2D convolutions.

The aforementioned nested design constitutes the complete forward-propagation process of the C3_Mamba module, detailed as follows:

Given the deep input feature tensor $X_{deep}\in {\mathbb{R}}^{B\times C\times H\times W}$, the system first employs two parallel 1 × 1 convolutions to decouple it into two independent information flows:

(1) $$Y_{1}=H_{mamba}({\mathrm{Conv}}_{cv1}(X_{deep}))$$

(2) $$Y_{2}={\mathrm{Conv}}_{cv2}(X_{deep})$$

Here, the main branch mapping function $H_{mamba}(\cdot )$ represents the core feature extractor composed of N cascaded Bottleneck_Mamba components, which is responsible for capturing global contextual dependencies within the deep feature space. The residual bypass (Skip Connection) $Y_{2}$ performs only a simple linear mapping, maximally preserving the low-level visual details and the original gradient flow, thereby effectively alleviating the vanishing gradient problem caused by the sequential stacking of complex operators in deep networks.

Ultimately, these two representations are concatenated along the channel dimension, and undergo cross-channel non-linear feature fusion via a terminal 1×1 convolution:

(3) $$Y_{final}={\mathrm{Conv}}_{cv3}(\mathrm{Concat}(Y_{1},Y_{2}))$$

Overall, this decoupled flow design not only allows the main branch to fully exploit the global perception advantages of Mamba, but also utilizes the bypass to stabilize the transmission of low-level features and gradients, thereby constructing a highly robust feature reconstruction module.

### 2.4. Sequential Modeling and Dynamic Selective Mechanism

The global perception capability of the main branch stems from the cascaded Bottleneck_Mamba components, as illustrated in Figure 4b. To achieve global receptive field coverage while constraining computational complexity, this component employs a “compression–global perception–expansion” residual bottleneck architecture. Given the input feature tensor $X_{in}$, it first undergoes channel compression via a 1×1 convolution. Subsequently, it is fed into the core MambaLayer spatial-to-sequence adapter to extract long-range dependencies. Finally, a terminal 1×1 convolution is utilized to restore the channel dimension, followed by the superimposition of the residual connection.

The feature evolution mechanism within the MambaLayer is illustrated in Figure 4c. To overcome the local receptive field constraints inherent in traditional two-dimensional convolutions, the system initially employs a spatial flattening and dimension transposition operation, denoted as F(·), to reconstruct the dimension-reduced 2D feature tensor $X_{in^{\prime }}$ into a 1D sequence $S\in {\mathbb{R}}^{B\times L\times C}$ (where the sequence length L=H×W). Subsequently, Layer Normalization (LayerNorm) is introduced to eliminate the internal covariate shift potentially induced by the serialization process, thereby ensuring numerical stability:

(4) $$S_{norm}=\mathrm{LayerNorm}(F(X_{in^{\prime }}))$$

To address the characteristics of complex backgrounds and minute defects inherent in UAV inspection images, this study introduces the Selective Mechanism—unique to State Space Models (SSMs)—into the Mamba Block. Specifically, the model first utilizes a 1D depthwise convolution (with a kernel size of $d_{conv}=4$) to perform local smoothing on the sequence $S_{norm}$, thereby extracting the spatial continuity prior $S_{local}$ of adjacent pixels. Subsequently, based on this local feature, the system dynamically generates the state transition step size Δ and the projection matrices B and C:

(5) $$\Delta =\mathrm{Softplus}(W_{\Delta }S_{local})$$

(6) $$B=W_{B}S_{local},\;\;\;C=W_{C}S_{local}$$

where $W_{\Delta }$, $W_{B}$ and $W_{C}$ are learnable weights. This dynamic mapping mechanism enables the model to effectively suppress redundant backgrounds and selectively amplify the feature responses of critical micro-abnormalities, such as missing split pins.

The feature evolution of the State Space Model originates from the continuous-time ordinary differential equation $\dot{h}(t)=Ah(t)+Bx(t)$. To adapt to the discrete nature of visual image sequences, the system employs the Zero-Order Hold (ZOH) rule to transform it into a discrete recursive form. Its state transition matrix $\bar{A}$ and input matrix $\bar{B}$ are approximated as follows:

(7) $$\bar{A}=\exp(\Delta A)$$

(8) $$\bar{B}={\left(\Delta A\right)}^{-1}(\exp(\Delta A)-I)\cdot \Delta B\approx \Delta B$$

Leveraging this discretization transformation, the inherently complex global contextual interactions can be rapidly accomplished through a hardware-aware parallel scan algorithm with a purely linear complexity of O(L):

(9) $$h_{t}=\bar{A}h_{t-1}+\bar{B}x_{t}$$

(10) $$y_{t}=Ch_{t}$$

Ultimately, the 1D sequence, having completed the global contextual interaction, is seamlessly reconstructed into a 2D spatial topology via linear projection and reshaping operations, denoted as $F^{-1}(\cdot )$. Combined with the external channel scaling and residual connection depicted in Figure 4b, the complete data flow of a single Bottleneck_Mamba component can be rigorously formulated as:

(11) $$X_{out}=X_{in}+{\mathrm{Conv}}_{1\times 1}(F^{-1}(\mathrm{SSM}(\mathrm{LayerNorm}(F({\mathrm{Conv}}_{1\times 1}(X_{in}))))))$$

Overall, this sequential modeling mechanism thoroughly bridges the architectural divide between the 1D state space and 2D visual features. By introducing virtually no additional computational overhead, it provides robust global representation support for the precise localization of micro-defects.

In summary, the proposed C3_Mamba module accomplishes highly efficient feature reconstruction through its macroscopic nested decoupling architecture and microscopic state space mechanism. The outer decoupling branch and residual connections consolidate low-level visual details and gradient flows, whereas the inner sequential modeling transcends the local limitations of traditional convolutions, endowing the network with long-range global perception capabilities. This “inner–outer synergistic” structure suppresses complex background interference in UAV inspections while providing precise global context for challenging micro-defects. It thus establishes a solid foundation for the synergistic multi-model detection framework with strict computational overhead control.

## 3. Experimental Setup

### 3.1. Experimental Dataset and Preprocessing

**Figure 5:**
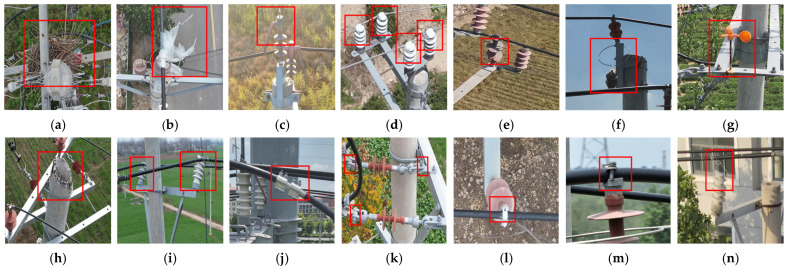
Visualization of 14 typical power line defect categories in the dataset. (**a**) Bird’s nest; (**b**) foreign object; (**c**) insulation sheath damage; (**d**) non-standard binding; (**e**) insulator damage; (**f**) missing arrester; (**g**) bird repeller damage; (**h**) damaged tower peak; (**i**) insulator inclination; (**j**) missing bolt; (**k**) clamp sheath detachment; (**l**) missing split pin; (**m**) bolt loosening; (**n**) missing tie wire.

To evaluate the effectiveness of the proposed framework in complex scenarios, a multi-scale power line defect dataset comprising UAV inspection images was constructed. The dataset contains 5137 images with an original resolution of 4000 × 3000 pixels, covering 14 typical power line defect categories. Specifically, the dataset includes BN (368), FO (269), ISD (126), NSB (58), ID (66), MA (199), BRD (496), DTP (879), II (428), CSD (845), MSP (668), MB (214), BL (188), and MTW (333). All defect samples were manually annotated using the Labeling tool and exported as standard VOC XML files. Figure 5 shows representative defect samples in the dataset, and Table 1 lists the names and abbreviations of all defect categories. During training, all images were uniformly resized and normalized to 640 × 640 pixels. Since MSP targets are extremely small in the original UAV images, the corresponding images were locally cropped into 640 × 640 patches before training to better preserve fine-grained defect details. The dataset was divided into training and validation sets at a ratio of 8:2. This fixed split was used for the main comparative experiments and ablation studies. All models involved in these experiments were trained and evaluated under the same data partition and training configuration.

**Figure 6:**
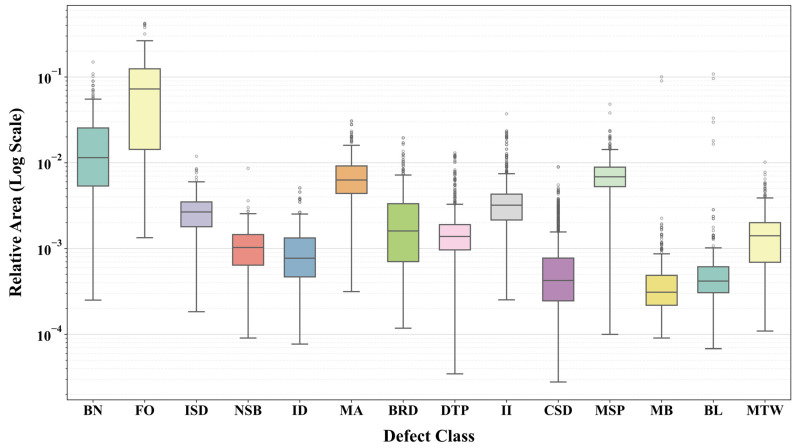
Multi-scale target area distribution of power line defects.

To describe the target-size distribution of the dataset, the relative target area was calculated as the ratio of the bounding-box area to the corresponding image area. As shown in Figure 6, different defect categories show obvious size differences on a logarithmic scale. FO and BN generally occupy larger image regions, whereas NSB, ID, CSD, MB, and BL are mainly distributed in smaller target-area ranges. However, the distributions of some categories overlap, indicating that the three defect groups cannot be divided only by the numerical value of the bounding-box area. For example, MSP shows a relatively larger target-area distribution because its original images were locally cropped into 640 × 640 patches before training, which reduces the image area and increases its relative target-area ratio. Nevertheless, MSP is still a fine-grained fastener-related defect in the original 4000 × 3000 UAV images. Therefore, the proposed grouping strategy considers not only the relative target area, but also the original visual scale, component semantics, and detection difficulty of each defect category.

Furthermore, this paper adopts a “divide-and-conquer” strategy, categorizing the 14 typical power line defects into three independent hierarchical levels based on their visual scales. Firstly, the macroscopic defect group (comprising BN, FO, ISD, and NSB) features large sizes and diverse morphologies. Processing these images independently enables the model to expand its receptive field and focus on overall contours, thereby avoiding entanglement in redundant local details. Secondly, the mesoscopic defect group (comprising ID, MA, BRD, DTP, II, and CSD) is typically attached to specific power fittings. Establishing an independent group for these guides the model to effectively learn the contextual positional relationships between “defects” and “components.” Finally, the microscopic defect group (comprising MSP, MB, BL, and MTW) occupies an extremely small pixel proportion and is highly susceptible to being submerged by cluttered field backgrounds. Isolating this group provides it with a dedicated feature ex-traction stream, allowing the network to concentrate exclusively on the detection of micro-defect features. Decoupled by visual scale, this hierarchical design alleviates feature learning conflicts between large and microscopic targets, laying a solid foundation for the subsequent multi-model collaborative detection framework.

### 3.2. Experimental Environment and Evaluation Metrics Algorithms

To ensure the fairness and complete reproducibility of the experimental results, the training and validation of all comparative models in this study were conducted within a unified computing environment. The software and hardware configuration details of the experimental platform are summarized in Table 2. To make the runtime condition clearer, the processor load, power consumption, and speed-limit-related information were also monitored during complete cascaded inference. The average GPU utilization was 11.11%, the average and peak power consumption were 132.10 W and 253.01 W, respectively, and the GPU operated under a 480 W power limit with an average SM clock of 2040 MHz. During network optimization, all models used the same initial learning rate, hyperparameter settings, and data partition to minimize the influence of external variables on model convergence and performance comparison.

To comprehensively evaluate the detection performance of the proposed framework under complex backgrounds and large scale variations, Precision, Recall, F1-score, and mean Average Precision (mAP) [31,32] were selected as the main evaluation metrics. Their definitions and descriptions are summarized in Table 3. Here, *TP*, *FP*, and *FN* denote true positives, false positives, and false negatives, respectively, and N denotes the number of defect categories.

In addition, mAP@0.5 and mAP@0.5:0.95 were used to evaluate the detection performance under different intersection over union (IoU) thresholds. mAP@0.5 reflects the basic recognition and localization ability of the detector when the IoU threshold is 0.5, while mAP@0.5:0.95 provides a stricter assessment of bounding-box localization quality across multiple IoU thresholds. Therefore, mAP@0.5:0.95 is useful for evaluating the precise localization capability of small and fine-grained defects, such as MSP.

## 4. Results and Discussions

### 4.1. Ablation Studies

To investigate the influence of the C3_Mamba insertion position, this study conducted ablation experiments by embedding the C3_Mamba module at different locations of the network using the ungrouped 14-class dataset under the same training settings. Specifically, four insertion strategies were compared: replacing the first two C3 modules in the backbone, replacing the last two C3 modules in the backbone, replacing the first two C3 modules in the neck, and replacing the last two C3 modules in the neck. The detailed results of the evaluation metrics are presented in Table 4.

The results show that the insertion position of C3_Mamba affects the detection performance. Among the four strategies, replacing the last two C3 modules in the backbone achieves the best overall results, with the highest precision, F1-score, mAP@0.5, and mAP@0.5:0.95. Compared with replacing the first two C3 modules in the backbone, the first two C3 modules in the neck, and the last two C3 modules in the neck, the final design improves mAP@0.5 by 2.3%, 2.8%, and 1.8%, respectively, and improves mAP@0.5:0.95 by 2.5%, 3.9%, and 2.5%, respectively. Precision is also increased by 3.4%, 2.6%, and 1.8%, respectively. Although the recall is slightly lower than that of the shallow-backbone variant, the higher F1-score and mAP values indicate that inserting C3_Mamba into the deeper backbone layers provides a better balance between detection accuracy and localization performance. This is because the deeper backbone contains richer semantic information, allowing C3_Mamba to capture global contextual features while preserving shallow spatial details and avoiding interference with neck feature fusion.

To evaluate the advantages of the core components within the framework, a systematic ablation study was conducted under a unified experimental setup. Specifically, the comparison schemes comprise: the traditional YOLOv5s baseline single model, the YOLOv5s multi-model detection based on the grouping strategy, the MS-Mamba network without grouping, and the ultimately proposed MS-Mamba.

**Figure 7:**
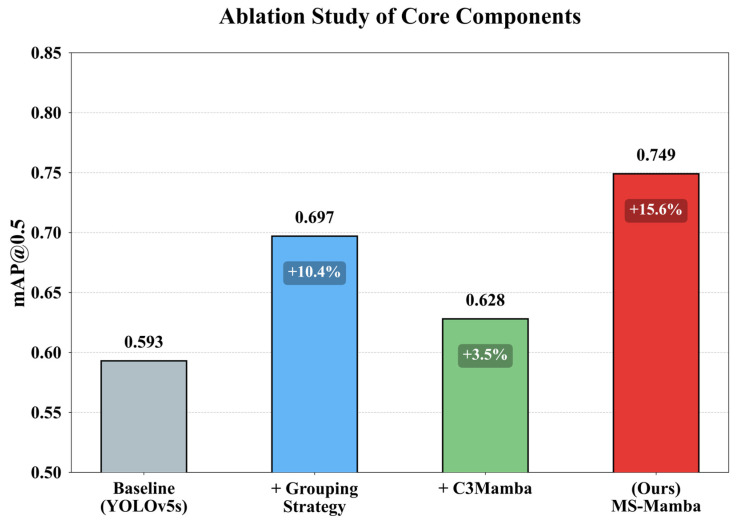
Ablation study of core components.

The comparison of the mAP@0.5 results for the four evaluation schemes is illustrated in Figure 7. When the original YOLOv5s baseline model is directly employed to detect the 14 defect categories of varying sizes, its mAP@0.5 metric reaches only 0.593. This indicates that a single convolutional network struggles to achieve satisfactory detection performance when processing targets with excessive scale discrepancies. Using this grouping strategy, the mAP@0.5 reached 0.697, a 10.4% absolute improvement over the un-grouped baseline. This demonstrates that processing targets of divergent sizes separately can effectively mitigate their mutual interference during the learning process. On the other hand, if only the C3_Mamba module is integrated into the YOLOv5s model for the simultaneous detection of all 14 defect categories, the mAP@0.5 rises to 0.628, an improvement of 3.5% over the YOLOv5s baseline. This substantiates the superiority of the proposed module in capturing global information. Furthermore, by integrating the grouping strategy with this core network to form the complete MS-Mamba, the mAP@0.5 peaks at 0.749, exhibiting a significant 15.6% performance improvement over the traditional YOLOv5s baseline single model. These results clearly demonstrate that the grouping strategy provides independent feature learning spaces for targets of varying scales, while the C3_Mamba module further enhances the model’s ability to capture faint defect features within these isolated spaces.

**Figure 8:**
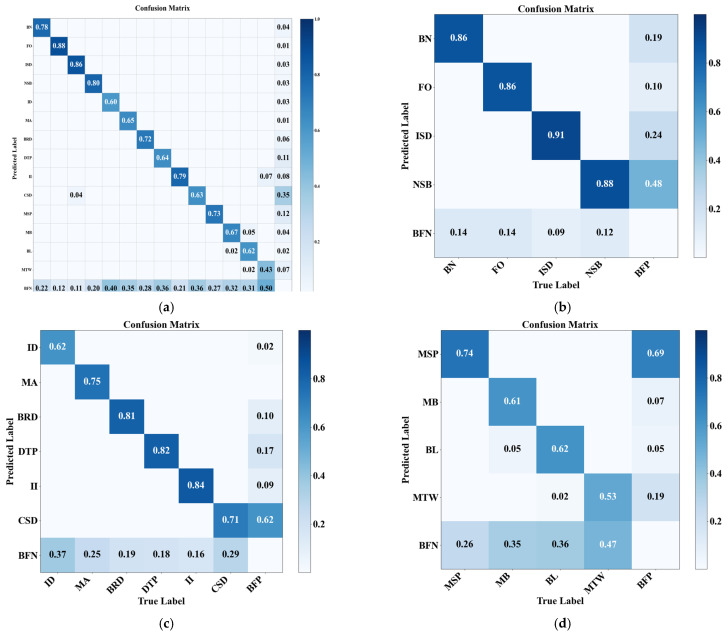
Confusion matrix comparison between unified detection and grouped detection. (**a**) Unified 14-class confusion matrix; (**b**) macroscopic defect group confusion matrix (BN–FO–ISD–NSB set); (**c**) mesoscopic defect group confusion matrix (ID–MA–BRD–DTP–II–CSD set); (**d**) microscopic defect group confusion matrix (MSP–MB–BL–MTW set).

To further investigate the intrinsic mechanisms driving the accuracy enhancement, this study generated and compared the confusion matrices of the ungrouped YOLOv5s baseline and the proposed MS-Mamba, as illustrated in Figure 8. It should be noted that the detector architecture remains unchanged across all settings; only the defect categories are decoupled into macroscopic, mesoscopic, and microscopic groups for independent evaluation. Compared with the unified detection results in Figure 8a, the grouped results in Figure 8b–d improve the diagonal values of most categories. For example, BN increases from 0.78 to 0.86, ISD from 0.86 to 0.91, MA from 0.65 to 0.75, DTP from 0.64 to 0.82, CSD from 0.63 to 0.71, and MTW from 0.43 to 0.53. These results indicate that the grouping strategy helps reduce cross-scale interference and enhances the recognition ability for most defect categories. At the same time, grouping also makes the differences in identifying defects of varying difficulty levels more apparent. FO defects often appear near conductors, towers, and complex backgrounds, where they can be confused with branches or shadows; thus, the diagonal value slightly decreases from 0.88 to 0.86. MB defects are very small and visually unclear, making them sensitive to shooting distance, occlusion, and nearby fittings, so the value decreases from 0.67 to 0.61. This overall elevation of the main diagonal values demonstrates that the grouping strategy effectively mitigates the mutual interference among targets of varying scales, thereby augmenting the discriminative ability of the model regarding defect categories.

To further validate the robustness of the proposed framework, a five-fold cross-validation was independently performed on each of the three sub-models re-sponsible for macroscopic, mesoscopic, and microscopic defect detection. The five-fold cross-validation in this study randomly divides the dataset of each category into five subsets. In each round of cross-validation, four subsets are used as the training set, while the remaining subset is used as the validation set, corresponding to an 8:2 split between the training and validation data. The detailed results of the evaluation metrics are presented in Table 5, Table 6 and Table 7.

As evident from the tables, the proposed method exhibits stable detection performance across defects of varying scales, with minimal overall fluctuations in the results of each group. This indicates that the grouped sub-models possess commendable robustness and generalization capabilities. Specifically, the mean mAP@0.5 values for the macroscopic and mesoscopic defect detection sub-models stabilized at 0.851 and 0.787, respectively. Notably, even for the microscopic sub-model subjected to the most severe background interference, its mean mAP@0.5 was maintained at 0.552, and its mean F1-score reached 0.594, exhibiting minimal numerical variance across the individual folds of the experiment. In conclusion, these results demonstrate that the proposed framework effectively mitigates overfitting and ensures reliable stability in complex detection scenarios.

### 4.2. Comparative Analysis of Different Models

To comprehensively evaluate the overall performance of the MS-Mamba network without grouping, this study compared it against four mainstream single-stage object detection algorithms: YOLOv3-tiny, YOLOv5s, YOLOv6s, and YOLOv8s. The specific evaluation results are presented in Table 8. As shown in the table, the proposed network achieves improvements across all five evaluation metrics compared to the other methods. Specifically, the Precision (P), Recall (R), and F1-score of the MS-Mamba network without grouping reach 0.639, 0.690, and 0.664, respectively. Regarding the mAP@0.5 metric, which reflects overall recognition ability, both YOLOv6s and YOLOv8s stagnate around 0.588, and the baseline model YOLOv5s achieves 0.593, whereas the proposed network successfully elevates this value to 0.628. In terms of the mAP@0.5:0.95 metric, which imposes more stringent requirements on bounding box localization accuracy, the lightweight YOLOv3-tiny scores only 0.312, and both YOLOv5s and YOLOv6s fall below 0.390. Even YOLOv8s, which performs relatively well among the baselines, only reaches 0.400. In contrast, the proposed model achieves a breakthrough to 0.418, representing a 3.9% improvement over the baseline YOLOv5s and demonstrating a significant leading advantage. This indicates that while capturing global information, the proposed network effectively preserves the edge details of the targets, thereby achieving more accurate localization when detecting micro-defects.

**Figure 9:**
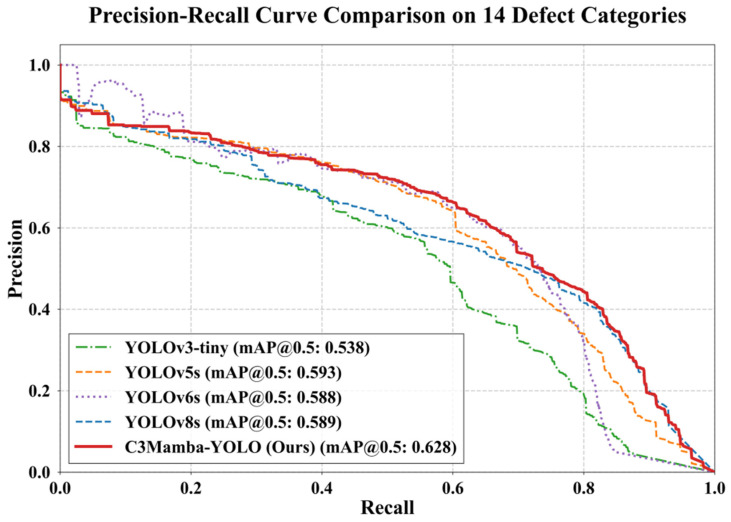
P-R curve comparison on 14 defect categories.

Furthermore, to evaluate the comprehensive performance of the proposed model in the mixed detection task involving 14 defect categories, this study compared the Precision–Recall (P-R) curves of YOLOv3-tiny, YOLOv5s, YOLOv6s, YOLOv8s, and the ungrouped MS-Mamba network, as shown in Figure 9. Overall, MS-Mamba achieves the largest enclosed area, with an mAP@0.5 of 0.628, indicating better comprehensive detection performance than the compared models. It can also be observed that MS-Mamba does not maintain the highest precision at every recall point. In some local intervals, especially in the low-recall region, several baseline models show comparable or slightly higher precision. This is mainly because low-recall predictions usually correspond to high-confidence detections, where the number of false positives is relatively small. However, as recall increases, most baseline curves decline more rapidly, indicating that they are more likely to introduce false detections when detecting more targets. In contrast, MS-Mamba maintains a smoother and more stable curve over a wider recall range, especially in the middle- and high-recall regions. This suggests that the proposed model achieves a better balance between recall improvement and false-positive suppression in complex backgrounds. Therefore, although MS-Mamba is not point-wise optimal over the entire P-R curve, it still obtains the best overall mAP@0.5 performance.

**Figure 10:**
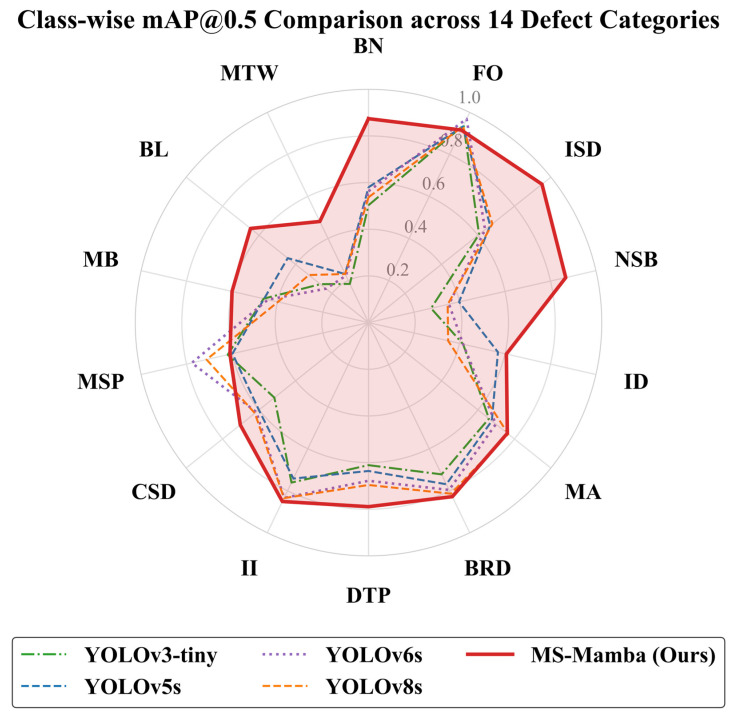
Impact of grouping strategy on class-wise performance.

To further analyze the detection and recognition capabilities of the framework for defects of varying scales, this study compared the results of the proposed MS-Mamba with the un-grouped mixed detection results of the 14 defect categories from four comparative algorithms: YOLOv3-tiny, YOLOv5s, YOLOv6s, and YOLOv8s. The mAP@0.5 metrics of each algorithm across the 14 specific categories are summarized in Table 9, and are visually represented in the radar chart shown in Figure 10.

As illustrated in Figure 10 and Table 9, the proposed MS-Mamba—represented by the red polygon in the chart—extends furthest outward along the axes of the vast majority of defect categories. This demonstrates that its mAP@0.5 is significantly superior to those of the comparative models, such as YOLOv3-tiny, YOLOv5s, YOLOv6s, and YOLOv8s. Consequently, this validates the robust generalization capability of the proposed method, proving that it is effective across a broad spectrum of categories rather than being restricted to merely a few specific ones.

Specifically, when confronted with microscopic defects such as MTW and BL, as well as categories like NSB, the four comparative algorithms—YOLOv3-tiny, YOLOv5s, YOLOv6s, and YOLOv8s—all exhibit pronounced inward indentations. Notably, the detection accuracy for MTW ranges merely from 0.184 to 0.231, and the recognition results for BL and NSB are similarly suboptimal. This demonstrates that when a traditional single network directly performs un-grouped mixed detection on 14 target classes characterized by extreme scale disparities, the features of large targets tend to obscure those of small targets. Consequently, this leads to a severe performance imbalance of the model across different categories.

Compared to the YOLOv5s baseline, the proposed MS-Mamba framework achieves substantial improvements in the aforementioned categories. Specifically, the detection accuracy for MTW is elevated to 0.481, representing an absolute increase of 25.0%, while the accuracy for BL reaches 0.647 with a 20.4% increment, and NSB achieves 0.867, yielding a notable 46.9% improvement. Furthermore, the contour of its radar chart appears significantly more well-rounded and balanced, indicating that the model experiences minimal performance fluctuations across diverse defect categories and possesses a more stable, comprehensive detection capability for all classes. These results demonstrate that the synergistic design of the adopted grouping strategy and the C3_Mamba module effectively mitigates the feature masking effect among multi-scale targets.

To evaluate the deployment efficiency of the proposed framework, this study further compared its processing speed and computational overhead with other detection models. As shown in Table 10, each MS-Mamba sub-model contains approximately 6.92 M parameters and 14.2 GFLOPs, which is slightly lower than YOLOv5s with 7.05 M parameters and 15.9 GFLOPs, indicating that each sub-model remains lightweight.

In practical deployment, the proposed framework adopts a priority-based cascaded inference strategy. The three sub-models are not necessarily activated for every input image. Once a valid defect is detected by a higher-priority sub-model, the result is directly used for report generation, and the remaining sub-models are no longer executed. To further evaluate the complete multi-model system, the complete cascade setting, in which all three sub-models are sequentially activated for one image, was also tested. In this setting, the complete system contains approximately 20.76 M parameters and requires about 42.6 GFLOPs. After excluding 20 warm-up images, the inference speed was evaluated on 1000 valid images with an input size of 640 × 640 and a batch size of 1. The complete system achieved an average inference time of 9.67 ms per image, corresponding to 103.38 FPS. These results demonstrate that the proposed framework achieves an effective balance between detection accuracy and deployment efficiency.

### 4.3. Detection Result Visualization

**Figure 11:**
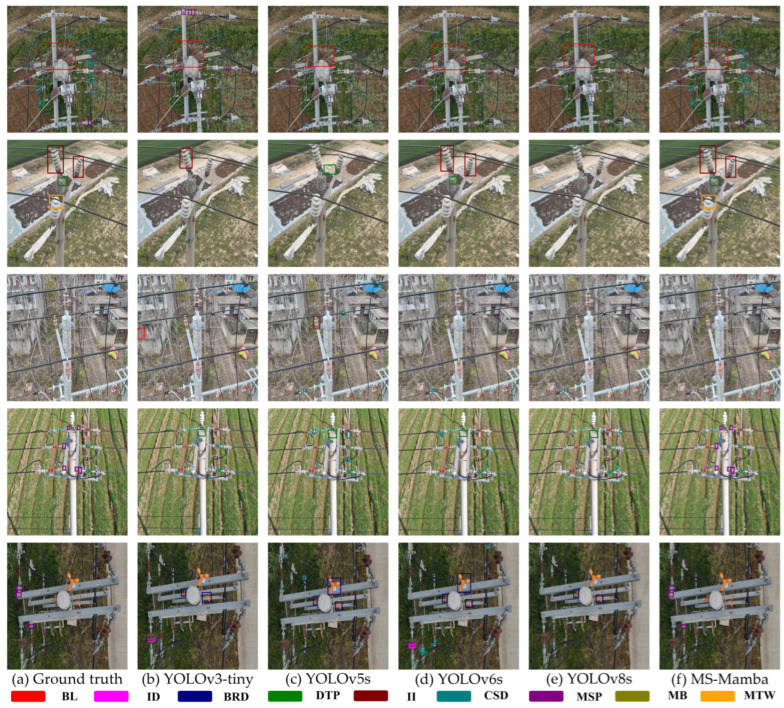
Visual comparison of detection results of different algorithms.

To evaluate the detection and recognition performance of the models more intuitively and further verify the effectiveness of the proposed method, representative samples were selected from the complex scenario test set. Inference was performed using YOLOv3-tiny, YOLOv5s, YOLOv6s, YOLOv8s, and the proposed MS-Mamba framework, respectively. A visual comparison of the detection results is presented in Figure 11. In the figure, different colors are utilized to distinguish and label various types of defects. Specifically, Figure 11a displays the ground truth annotations for each test sample, serving as the benchmark for subsequent comparison. The remaining subfigures present the inference results of different algorithms as follows: (b) YOLOv3-tiny, (c) YOLOv5s, (d) YOLOv6s, (e) YOLOv8s, and (f) MS-Mamba.

As can be observed from Figure 11, when defects with extreme scale discrepancies appear simultaneously within the same frame (for instance, the large BN and the microscopic defect on the power fitting in the first row), traditional comparative algorithms such as YOLOv3-tiny, YOLOv5s, YOLOv6s, and YOLOv8s are highly susceptible to missing small targets because the large targets are overly conspicuous. In contrast, the proposed model not only precisely localizes macroscopic defects but also detects microscopic defects that are easily overlooked. This improvement is primarily attributed to the physical attribute grouping strategy, which processes targets of varying sizes separately, effectively preventing the features of large targets from masking the faint features of small targets.

Furthermore, when dealing with extremely small targets concealed within complex field backgrounds (such as the trees in the third row and the farmland in the fourth row), traditional single networks frequently suffer from missed detections or deviated bounding box locations. Conversely, for these error-prone hard samples, the proposed model remains capable of generating highly accurate and tightly fitting prediction boxes. This is credited to the dynamic selection mechanism of the C3_Mamba module, which assists the network in automatically filtering out redundant background interference, enabling the model to concentrate its attention on genuine defect features. These visual results intuitively demonstrate that the proposed method possesses exceptional detection capabilities in complex inspections.

### 4.4. System Implementation and Validation

**Figure 12:**
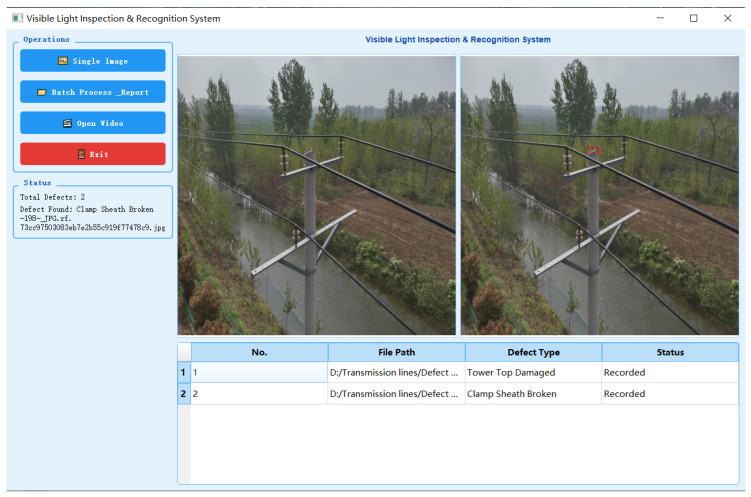
Visible light inspection and recognition system.

To verify the engineering applicability of the proposed framework, this study integrated and deployed the MS-Mamba framework into an independently developed visible-light intelligent inspection and recognition system, as illustrated in Figure 12. The left-hand operation panel of the system integrates three primary functional modules—single image detection, batch image processing and report generation, and video detection—to accommodate diverse inspection requirements. In the central section of the interface, the system adopts a dual-view design, with the left side displaying the original input image and the right side presenting the defect image annotated with predicted bounding boxes. Furthermore, a dynamic data log table is situated at the bottom of the interface, capable of recording the defect count, folder paths, and defect names in real-time, thereby achieving visual traceability throughout the defect detection and recognition process.

**Figure 13:**
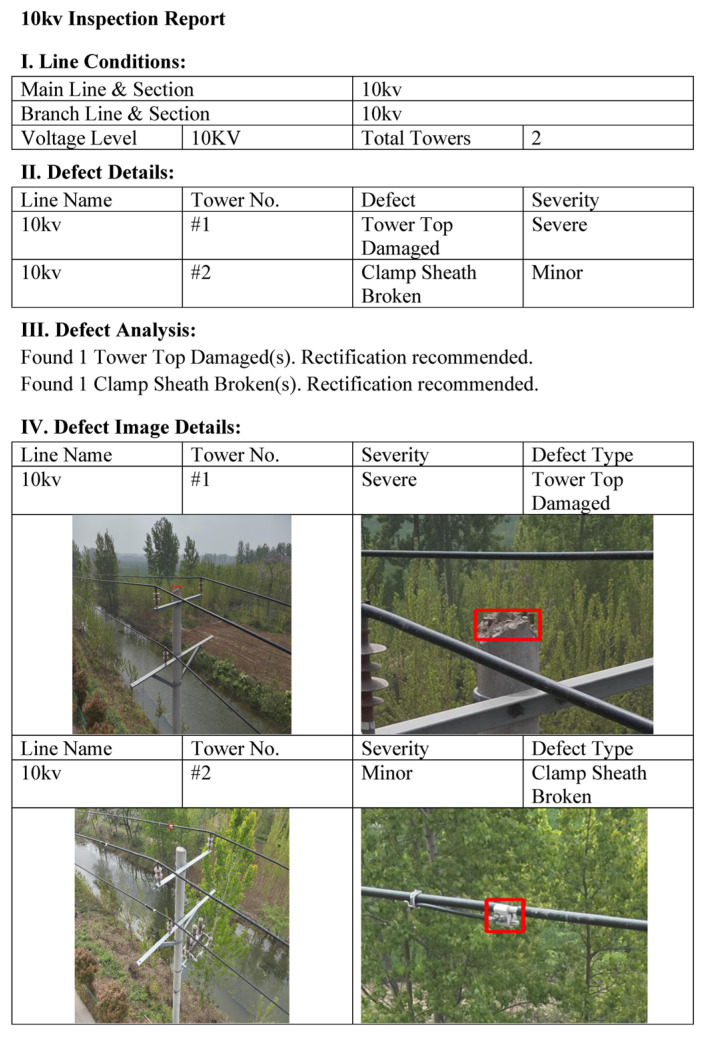
Inspection report.

To enhance the management efficiency of large-scale inspection tasks, the system further incorporates an automated inspection report generation module. Upon initiating the batch processing mode, the system automatically traverses the directories containing the original inspection images and simultaneously invokes the three pre-trained grouped sub-models to perform inference. Following the completion of the inference process, the system automatically generates a standardized inspection report, as illustrated in Figure 13. This report encompasses specific details regarding the inspected power lines, including the voltage level, tower number, defect name, and severity. Concurrently, the report automatically appends the complete defect image annotated with predicted bounding boxes, alongside a locally magnified cropped image of the defective region, thereby providing an intuitive visual reference for subsequent maintenance and repair operations.

To further validate the proposed method in terms of processing time, false detections, missed detections, and practical defect screening capability, we conducted verification experiments on a highly simulated real-scenario dataset. The dataset contains 741 images and was designed to approximate practical engineering conditions in terms of scene complexity, defect types, and background interference. The detailed results are presented in Table 11.

As shown in Table 11, we conducted engineering validation using 741 highly realistic inspection images, including 578 defective images and 163 defect-free images. The system correctly identified 558 defective images and 133 defect-free images, with 20 missed detections and 30 false detections. The missed detection rate was only 3.46%, indicating strong defect screening ability in near-realistic scenarios. Although the false detection rate for defect-free images reached 18.40%, these false alarms can be further filtered through manual review of the automatically generated inspection reports. Therefore, while effectively reducing missed detections, the proposed system provides traceable and reviewable results for maintenance decision-making.

Overall, the proposed end-to-end workflow, from batch image import to standardized report generation, reduces manual verification time and subjective errors. The quantitative validation further confirms that the MS-Mamba-based inspection system achieves a low missed detection rate in highly simulated scenarios and provides traceable review evidence for manual final decision-making. These results demonstrate its practical value and deployment feasibility in large-scale power grid inspection tasks.

## 5. Conclusions

This study proposed a Multi-Scale Mamba Framework (MS-Mamba) for multi-defect detection in power line inspection images. The main conclusions are summarized as follows:

1. Through the synergy effect of the physical attribute grouping strategy and the C3_Mamba module, the proposed MS-Mamba framework achieved an mAP@0.5 value of 0.749 in detecting 14 types of defects, resulting in an improvement of 15.6% compared to the YOLOv5s baseline.
2. The introduced global dynamic selection mechanism significantly improved the ability to detect the smallest defects against complex backgrounds. In tests evaluating the MS-Mamba network without grouping, the rigorous evaluation metric mAP@0.5:0.95 reached a value of 0.418, which corresponds to a 3.9% improvement over YOLOv5s. It also showed excellent stability in the high recall range of the P-R curve.
3. In terms of model efficiency, each MS-Mamba sub-model contains approximately 6.92M parameters and 14.2 GFLOPs, reducing the parameter count and computational cost by 0.13M and 1.7 GFLOPs, respectively, compared with YOLOv5s. Under the complete cascaded inference setting, the system achieved 9.67 ms per image and 103.38 FPS, indicating a practical balance between detection accuracy and inference efficiency.
4. An end-to-end inspection workflow was developed by integrating defect detection, result visualization, defect archiving, and report generation. This workflow improves the automation level of power line inspection and provides practical support for engineering-oriented defect detection.

Future work will focus on further extending the proposed framework from inspection-image-based validation to full-cycle field-line deployment.

Overall, the proposed MS-Mamba framework provides an effective solution for accurate and efficient multi-defect detection in complex power line inspection scenarios.

## Figures and Tables

**Table 1:** Defect type and abbreviation.

Abbreviation Symbols	Defect Type	Abbreviation Symbols	Defect Type
BN	Bird’s nest	II	Insulator inclination
FO	Foreign object	CSD	Clamp sheath detachment
ISD	Insulation sheath damage	MSP	Missing split pin
NSB	Non-standard binding	MB	Missing bolt
ID	Insulator damage	BL	Bolt loosening
MA	Missing arrester	MTW	Missing tie wire
BRD	Bird repeller damage	BFN	Background FN
DTP	Damaged tower peak	BFP	Background FP

**Table 2:** Experimental platform configuration.

Platform	Configuration
Operating system	Ubuntu 22.04
Graphics processor	NVIDIA GeForce RTX 3090Ti
GPU accelerator	CUDA 12.1 and cuDNN 9.1.0
Deep learning framework	Pytorch version 2.5.1
Programming language	Python version 3.10

**Table 3:** Evaluation metrics used in this study.

Metric	Definition	Description
Precision	$\frac{TP}{TP+FP}$	Measures the proportion of correctly detected defects among all predicted defects.
Recall	$\frac{TP}{TP+FN}$	Measures the proportion of correctly detected defects among all actual defects.
F1-score	$\frac{2\times Precision\times Recall}{Precision+Recall}$	Provides a balanced evaluation between Precision and Recall.
mAP	$\frac{1}{N}\sum_{i=1}^{N}AP_{i}$	Measures the average detection performance over all defect categories.

**Table 4:** Ablation results of different C3_Mamba insertion positions.

C3_Mamba Insertion Position	P	R	F1	mAP@0.5	mAP@0.5:0.95
First two C3 modules in the backbone	0.605	0.695	0.647	0.605	0.393
First two C3 modules in the neck	0.613	0.674	0.642	0.600	0.379
Last two C3 modules in the neck	0.621	0.691	0.654	0.61	0.393
Last two C3 modules in the backbone (Ours)	0.639	0.690	0.664	0.628	0.418

**Table 5:** Macro-defect group evaluation indicators.

Fold Number	P	R	F1	mAP@0.5	mAP@0.5:0.95
Fold-1	0.953	0.809	0.875	0.864	0.527
Fold-2	0.946	0.810	0.873	0.848	0.516
Fold-3	0.945	0.813	0.874	0.863	0.544
Fold-4	0.895	0.741	0.811	0.779	0.475
Fold-5	0.937	0.819	0.874	0.902	0.579
Average	0.935	0.798	0.861	0.851	0.528

**Table 6:** Mesoscopic defect group evaluation indicators.

Fold Number	P	R	F1	mAP@0.5	mAP@0.5:0.95
Fold-1	0.875	0.745	0.805	0.775	0.567
Fold-2	0.885	0.778	0.828	0.799	0.590
Fold-3	0.881	0.756	0.814	0.794	0.581
Fold-4	0.877	0.782	0.827	0.810	0.599
Fold-5	0.878	0.716	0.789	0.756	0.546
Average	0.879	0.755	0.812	0.787	0.577

**Table 7:** Microscopic defect group evaluation indicators.

Fold Number	P	R	F1	mAP@0.5	mAP@0.5:0.95
Fold-1	0.671	0.556	0.608	0.568	0.269
Fold-2	0.636	0.510	0.566	0.498	0.242
Fold-3	0.604	0.575	0.589	0.549	0.262
Fold-4	0.650	0.564	0.604	0.562	0.290
Fold-5	0.588	0.621	0.604	0.584	0.312
Average	0.630	0.565	0.594	0.552	0.275

**Table 8:** Performance comparison of different object detection algorithms.

Method	P	R	F1	mAP@0.5	mAP@0.5:0.95
YOLOv3-tiny	0.579	0.603	0.591	0.538	0.312
YOLOv5s	0.623	0.665	0.643	0.593	0.379
YOLOv6s	0.599	0.670	0.626	0.588	0.384
YOLOv8s	0.578	0.638	0.607	0.589	0.400
MS-Mamba	0.639	0.690	0.664	0.628	0.418

**Table 9:** mAP@0.5 index for 14 types of defects.

Class 14 Defects	YOLOv3-Tiny	YOLOv5s	YOLOv6s	YOLOv8s	MS-Mamba
BN	0.503	0.580	0.563	0.537	0.874
FO	0.939	0.933	0.969	0.934	0.916
ISD	0.606	0.667	0.639	0.679	0.951
NSB	0.275	0.398	0.354	0.348	0.867
ID	0.416	0.569	0.416	0.350	0.606
MA	0.660	0.676	0.698	0.766	0.761
BRD	0.722	0.769	0.796	0.814	0.828
DTP	0.611	0.636	0.678	0.696	0.789
II	0.761	0.742	0.834	0.835	0.851
CSD	0.516	0.601	0.615	0.623	0.703
MSP	0.619	0.598	0.777	0.712	0.609
MB	0.461	0.459	0.442	0.398	0.600
BL	0.264	0.443	0.233	0.327	0.647
MTW	0.184	0.231	0.223	0.231	0.481

**Table 10:** Comparison of computational complexity among different models.

Model	Layers	Params(M)	GFLOPs(G)
Yolov3-tiny	38	8.70	12.9
Yolov5s	157	7.05	15.9
Yolov6s	504	18.51	44.87
Yolov8s	225	11.14	28.7
MS-Mamba	197	6.92	14.2

**Table 11:** Quantitative validation results in simulated inspection scenarios.

Evaluation Item	Result
Total number of inspection images	741
Number of defective images	578
Number of defect-free images	163
True positives	558
True negatives	133
False positives	30
False negatives	20
False detection rate	18.40%
Missed detection rate	3.46%

## Data Availability

Data will be made available on request.

## Abbreviations

The following abbreviations are used in this manuscript:

YOLO	You Only Look Once
UAV	Unmanned Aerial Vehicle
MS-Mamba	Multi-Scale Mamba Framework
C3	CSP Stage Block with 3 Convolutions
MID-Net	Multiscale Insulator Defect Detect Network
KCDet	Keypoint Clustering Detector
FS-SSD	Fusion and Scaling-based Single Shot Detector
Faster R-CNN	Faster Region-based Convolutional Neural Network
Wiou	Wise Intersection over Union
PSTL-Net	Patchwise Self-texture-learning Network
FPF	Focused Perception Framework
YOLO-DTAD	You Only Look Once–Dynamic Task Alignment Detection
LA-YOLO	Lightweight Adaptive You Only Look Once
C2f	CSP Stage Block with 2-flows
MBLEConv	Mobile Bottleneck Lite-effective Convolution
CNN	Convolutional Neural Network
SSM	State Space Model
CSPNet	Cross-Stage Partial Network
mAP	Mean Average Precision
Params	Parameters
GFLOPS	Giga Floating-point Operations Per Second
